# Supplementary material for: Aryl Hydrocarbon Receptor (AhR) Activation by 2,3,7,8-Tetrachlorodibenzo-p-Dioxin (TCDD) Dose-Dependently Shifts the Gut Microbiome Consistent with the Progression of Non-Alcoholic Fatty Liver Disease
Source: Int J Mol Sci. 2021 Nov 18;22(22):12431. doi: 10.3390/ijms222212431 (PMC8625315; doi:10.3390/ijms222212431)
Supplement: Supplementary file 1 [file ijms-22-12431-s001.zip › IMJS_supplemental_figures-1 - TZ 102721 - RF 102721.pdf]

Supplemental Material

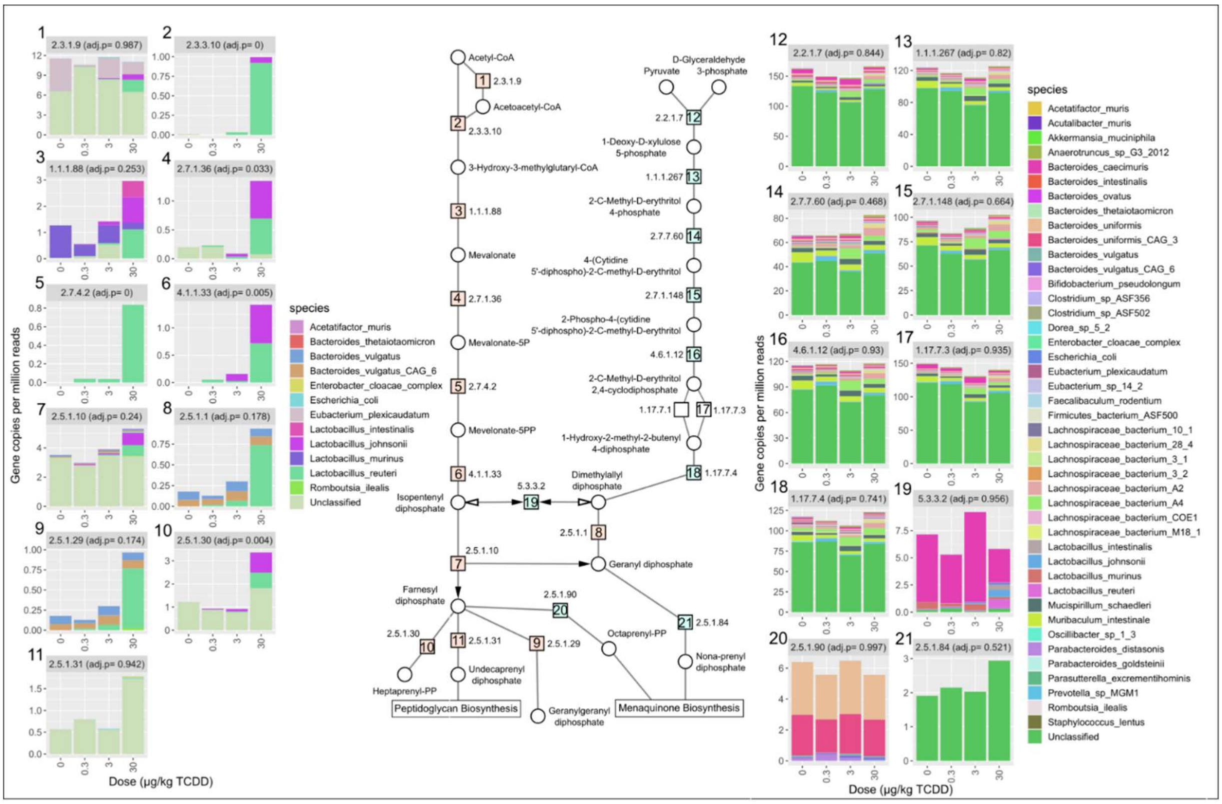

Figure S1. Stacked bar plots of annotated EC number mean relative abundance and classified species involved in isoprenoid biosynthesis in cecum of TCDD-exposed mice. Male C57BL6 mice were orally gavaged with sesame oil vehicle or 0.3, 3, or 30 µg/kg TCDD every 4 days for 28 days (n=3). Numbers beside each bar plot correlate with numbered EC number provided in the center schematic of isoprenoid biosynthesis. Adjusted *p*-values (adj. p) were determined by the Maaslin2 R package.

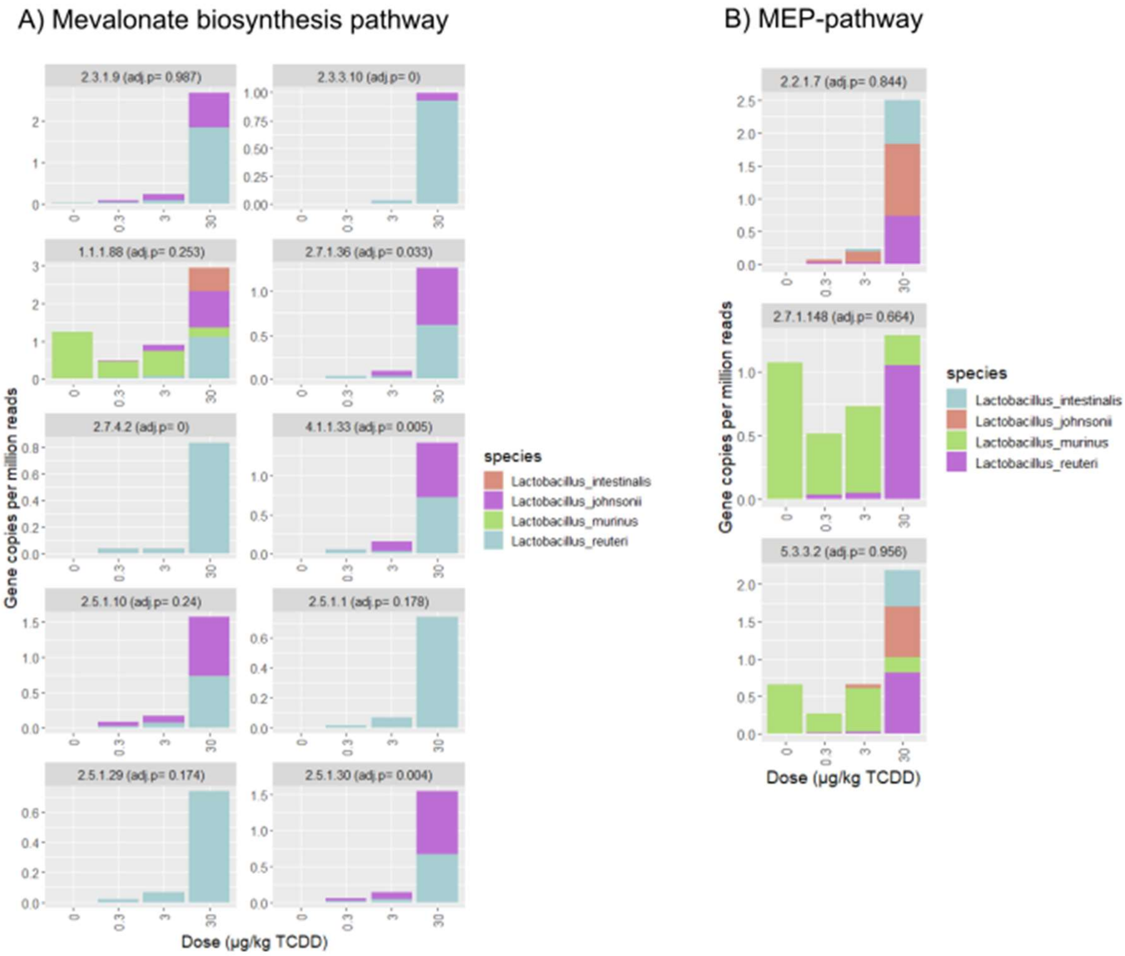

Figure S2. Stacked bar plots of mean relative abundance of annotated EC numbers and classified to Lactobacillus species involved in isoprenoid biosynthesis in cecum of TCDD-exposed mice. Male C57BL6 mice were orally gavaged with sesame oil vehicle or 0.3, 3, or 30 µg/kg TCDD every 4 days for 28 days (n=3). Adjusted *p*-values (adj. p) were determined by the Maaslin2 R package and denote significance.

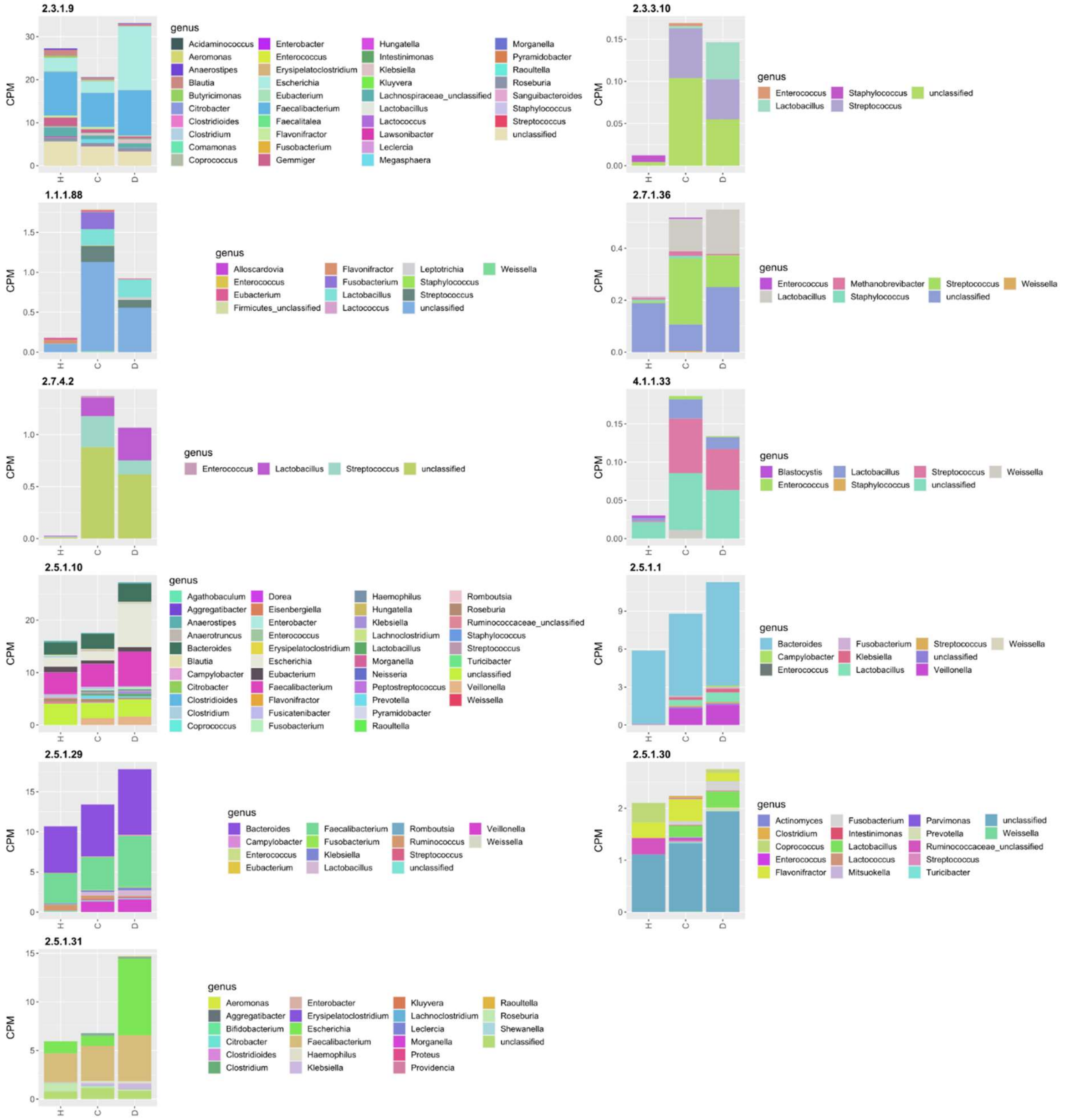

Figure S3. Stacked bar plots of annotated EC number mean relative abundance classified to respective genus and involved in mevalonate-dependent isoprenoid biosynthesis in cirrhosis patients. Fecal metagenomic samples were from healthy (H, n=52), compensated (C, n=48) or decompensated (D, n=44) diagnostically defined groups.
